# Cytotoxic effects on human dental pulp stem Cells after exposure to adhesive bonding agents

**DOI:** 10.1590/0103-6440202405529

**Published:** 2024-06-24

**Authors:** Antonella Castellanos Caroprese, Alexa Nicolle Chinchilla Navarrete, Sandra Janeth Gutiérrez Prieto, Jean Carlos Villamil, Octavio Alejandro Castañeda Uribe, Juan Carlos Salcedo Reyes, And Henry Alberto Méndez Pinzón

**Affiliations:** 1 Centro de Investigaciones Odontológicas, Facultad de Odontología, Pontificia Universidad Javeriana, BogotáD.C., Colombia; 2 Escuela de Ingeniería, Ciencia y Tecnología, Universidad del Rosario, BogotáD.C., Colombia; 3 Grupo de Películas delgadas y Nanofotónica, Departamento de Física, Pontificia Universidad Javeriana, BogotáD.C., Colombia

**Keywords:** Cytotoxicity, Cell viability, Raman Spectroscopy, Human Dental Pulp Stem Cells, self-etching adhesive bonding agents

## Abstract

Studies regarding cytotoxic effects attributed to the use of adhesive bonding agents on pulp tissue are not conclusive. To point out whether these materials are safe for clinical use, in vivo exposure of dental pulp to adhesive bonding agents was simulated using an experimental setup in which Human Dental Pulp Stem Cells (hDPSC) are exposed to the action of two kinds of adhesives: self-etching adhesives and two-step bonding agents through a dentine barrier. Cytotoxic effects on these cells were evaluated by MTT assay protocol and fluorescence microscopy, and their results were contrasted to those obtained through Raman spectra taken on single hDPSCs. Overall, no significant cytotoxic effects were observed by combining all the techniques, and cell viability close to 90% was achieved for a dentine barrier of at least 1 mm thick. Moreover, Raman spectroscopy was able to detect structural DNA damage in some dental pulp cells when exposed to two-step bonding agents, suggesting that this technique could be considered a complementary tool with the potential to evaluate cell toxicity beyond cell viability.

## Resumo

Os estudos sobre os efeitos citotóxicos atribuídos ao uso de agentes de união adesivo no tecido pulpar não são conclusivos. Para determinar se esses materiais são seguros para uso clínico, a exposição in vivo da polpa dentária a agentes de união adesiva foi simulada por meio de uma configuração experimental na qual as células-tronco da polpa dentária humana (hDPSC) são expostas à ação de dois tipos de adesivos: adesivos autocondicionantes e agentes de união de duas etapas por meio de uma barreira de dentina. Os efeitos citotóxicos nessas células foram avaliados pelo protocolo de ensaio MTT e microscopia de fluorescência, e seus resultados foram contrastados com os obtidos por meio de espectros Raman obtidos em hDPSCs individuais. De modo geral, não foram observados efeitos citotóxicos significativos com a combinação de todas as técnicas, e a viabilidade celular próxima a 90% foi obtida para uma barreira de dentina de pelo menos 1 mm de espessura. Além disso, a espectroscopia Raman foi capaz de detectar danos estruturais ao DNA em algumas células da polpa dentária quando expostas a agentes de colagem de duas etapas, sugerindo que essa técnica poderia ser considerada uma ferramenta complementar com potencial para avaliar a toxicidade celular além da viabilidade celular.

## Introduction

Governed by the concept of minimal intervention, the use of adhesives implemented by Michel G. Buonocore has changed the concept of cavity design in dental praxis, reducing the size of dental surgery areas by avoiding the unnecessary elimination of dental tissue ^1^. Accordingly, components and techniques used in adhesive systems have evolved with time, reducing steps in their application. That is the case of self-etching adhesive bonding agents, that promote adhesion without remotion of the smear layer, originating an interfacial hybrid layer in just one step. Such adhesives are based on the selective acid etching technique, recommended for deep cavities near the pulp due to their reduced working times and post-operative sensitivity ^2^ ^,^ ^3^.

Despite the improvement of self-etching adhesive bonding agents, cytotoxic studies performed by traditional techniques like Fluorescence microscopy and MTT assay have detected non-polymerized residual monomers of the components bis-GMA, HEMA, TEGDMA, UDMA, glutaraldehyde, and camphorquinones in cell cultures ^4^ ^,^ ^5^ ^,^ ^6^. Residual HEMA monomers, for instance, can reach pulp tissue through dentin tubules, reducing cell metabolism or causing irreversible damage ^7^. However, there are still controversial in vivo and in vitro studies reporting no harmful effects on pulp tissue attributed to those components. Therefore, it is extremely important to design experiments for assessing in vivo cell viability and toxicity of pulp tissue, after being exposed to extensively used adhesives, as close as possible to the conditions of dental praxis ^8^.

Odontoblasts are the first kind of pulp cells to react to exogen stimuli after the application of different dental materials ^9^. However, they are difficult to grow due to their postmitotic phenotype, and hence, biochemical or toxicological investigations on them are limited ^10^ ^,^ ^11^. To overcome this situation, cytotoxicity effects have been conducted on primary gingival fibroblasts ^12^ ^,^ ^13^, Human Mesenchymal Stem Cells, primary mouse cells, and immortalized cell lines ^14^. In this work, Human Dental Pulp Stem Cells (hDPSC) were chosen for several reasons: low mortality rate, high capacity of differentiation and proliferation, excellent adhesion, ease of obtaining extraction in cell culture ^15^, and their ability to preserve the original cell characteristics without genetic changes after proliferation. Reparation of dentine and enamel is the main function of hDPSC, allowing for the simulation of clinic conditions to check the safety of the employment of dental materials ^16^ ^,^ ^17^ ^,^ ^18^.

One of the standard methods for evaluating cytotoxicity by assessing cell metabolic activity is the MTT assay. The tetrazolium dye MTT 3-(4,5-dimethylthiazol-2-yl)-2,5-diphenyltetrazolium bromide is captured and reduced by oxidoreductase enzymes like mitochondrial succinic dehydrogenase to its insoluble compound formazan, which has a purple color, allowing their quantification by colorimetric methods. The cell capacity for reducing MTT is an indicator of mitochondrial integrity, and their functional activity is interpreted as a measurement of cell viability. However, this technique requires many steps for sample preparation using several chemical compounds in the process. In addition, it demands the employment of complementary instrumentation for checking the results, traditionally fluorescence microscopy or flow cytometry, introducing additional steps, time, and costs ^19^ ^,^ ^20^.

Due to the limitations of MTT assay, Raman spectroscopy was proposed in this study to evaluate their application as a complementary tool for assessing cell viability and even their degree of toxicity. This is an optical technique based on the radiation-matter interaction of a dispersed monochromatic laser light, providing information about the vibrational properties of molecules of the substances under examination. In this sense, the "fingerprint" of substances enables their qualitative and quantitative estimation. When a low optical power incident laser light is applied, it is possible to measure samples without changing their functionality and molecular structure, making Raman Spectroscopy a non-destructive analysis technique of samples that does not require many steps for their preparation. In their microscopic configuration, an incident laser beam of a few microns diameter is directly applied to individual cells and light dispersed from different constituents of the cell can be analyzed, providing differentiated information. All these features, their relatively low cost, and their capability for analysis independently of another technique give Raman Spectroscopy a huge potential for diagnosis of cell viability ^21^ ^,^ ^22^. Therefore, the capability of Raman Spectroscopy for detecting cytotoxic effects induced on hDPSC by exposure to two kinds of self-etching adhesive bonding agents was evaluated in the frame of this work. Surface-Enhanced Raman Scaterring (SERS) effect was used to amplify the Raman signal from individual cells ^23^ ^,^ ^24^. Their results were contrasted with those obtained by Fluorescence microscopy and MTT assay protocol.

## Materials and methods

### Experimental setup

An experimental setup resembling the exposure of pulp tissue to adhesive agents in clinical practice was designed. Dentine prevents direct contact with pulp tissue when adhesives are applied in a dental cavity. However, dentine has microtubules through which chemicals can reach the pulp.

**Figure 1:**
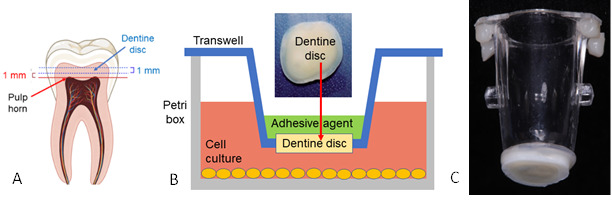
Experimental design for simulating exposure of dental pulp to adhesive agents in clinical practice: a) Diagram of cutting area of dentin discs in molars. b) a Real dentine disc image and their placement as a barrier between adhesive agents and cell culture and c) a Picture showing a 12.0 mm diameter dentin disc fixed to the transwell.

To simulate the dentine wall in dental deep cavities, a dentine disc of nearly 1.0 mm thickness was cut from a region above the pulp horns of a molar (Figure 1a). In the experiment, the dentine disc replaces the membrane on the bottom of Corning*®* Transwell*®* cell culture inserts, acting as a barrier between adhesive agents and cell culture (Figures 1b and 1c). Thus, Poietics™ hDPSC from Lonza*®* protected by a 1mm-thick dentine barrier were exposed to the adhesive agents Single Bond Universal (3M™), Opti bond All In One (Kerr™) and Adper single bond (3M™) for 24 hours.

### Sample preparation

**Figure 2:**
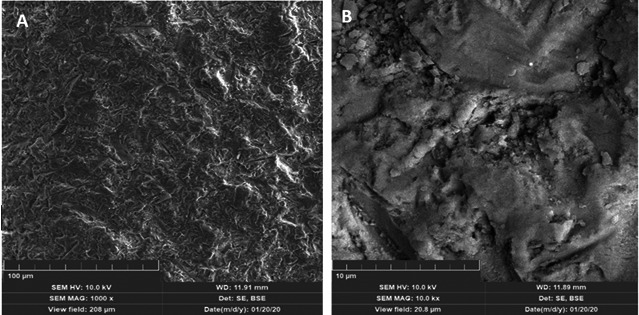
Scanning Electron Microscopy (SEM) images of a dentine disc: a) Smear layer on the occlusal surface (1000X) and b) Under 10.0 kX. The structure and morphology of dentine debris within the smear layer are observed.

Once patients signed the consent for organ donation, 36 prescribed molars were extracted and kept in distilled water at 4ºC. Prior to obtaining dentine discs, periapical radiographs with standard angle were taken. The cut was performed 1.0 mm above pulp horns, a dental region with a high density of microtubules (30.000 to 50.000) ^25^, as observed on SEM images taken by a Tescan Lyra3 GM in Figure 2.

For cutting, a calibrated microtome ISOMET*®* 1000 precision sectioning saw (Buehler) was used to resemble the specific characteristics of deep cavities close to the pulp chamber, where the use of self-etching adhesive bonding agents is indicated. Dentine discs were placed instead of the 12.0 mm diameter membrane of Corning*®* Transwell*®* polycarbonate membrane cell culture inserts.

### Cell culture

**Figure 3:**
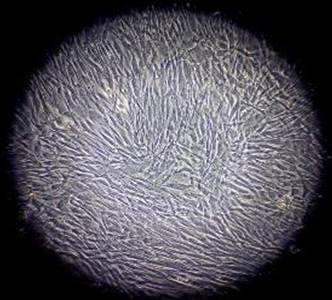
Monolayer of hDPSC under an inverted microscope (40x).

Poietics™ Human Dental Pulp Stem Cells (hDPSC) from Lonza*®* were purchased and processed according to the instructions. Two cell cultures of around 250.000 Cells were kept. After sowing with a 5 mL culture medium, it was changed the following day. Since then, the culture medium has changed every couple of days. Cell subculture lasted nearly a week until a monolayer with a confluence between 80 and 90% was reached. Mitotic features were observed under the optical microscope (Figure 3).

### Preparation of SERS substrates

**Figure 4:**
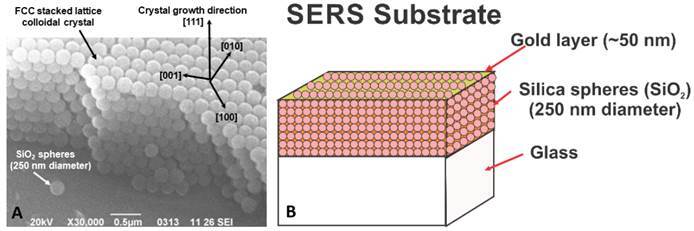
SERS substrates elaborated by deposition of a metallic layer on colloidal crystals (CC). a) SEM image of FCC colloidal crystal grown by stacked 250 nm Silica spheres. b) Structure of the SERS substrate with a 50 nm thickness gold layer on top (Ref. ^24^).

Usually, Raman signal from organic samples is weak and noisy and Self-Enhanced Raman Scattering (SERS) must be used to amplify dispersed radiation in many orders of magnitude. The SERS effect is based on the resonant interaction of radiation at a metal-organic interface, resulting in an amplification of dispersed Raman signal from organic molecules absorbed to metallic surfaces with periodic roughness at a nanometric scale ^23^ ^,^ ^24^. Therefore, the periodicity of a colloidal crystal (CC) covered by a thin metallic layer, has been used for elaborating suitable SERS substrates, able to provide Raman signal steaming from individual cells focused under an optical microscope (Figure 4) ^23^.

### Sewing hDPSC on SERS substrates

After reaching a confluence higher than 80% medium culture is thrown away. Cell debris is removed by rinsing with 5 mL buffered saline solution HEPES-BSS (LONZA). Detachment of cell culture is made in the incubator by adding 2 mL trypsin/EDTA solution for 5 minutes (LONZA). After detached, 4 mL trypsin neutralizing solution TNS (LONZA) is added. The resulting suspension is centrifugated at 2200 rpm and 4ºC for 5 minutes. The supernatant is discarded, and the resulting pellet is suspended in 1 mL of cell culture medium and a live cell count is performed in the Neubauer chamber. In a laminar flow chamber, nearly 150.000 cells were sewed on SERS substrates, previously sterilized with Ethylene oxide and under UV radiation for one hour. The substrates were placed on the bottom, and immersed within 2 mL medium culture contained on each one of 12 wells in Corning*®* culture cell dishes. The cell culture sewn on SERS substrates will be used only to perform Raman measurements. That kind of substrate is not needed for Fluorescence and MTT tests.

### Design of the experiment

Based on the setup shown in Figure 1b, hDPSC were exposed to three different adhesive agents: Group 1 - Single bond universal 3M™ (SBU), Group 2 - Opti bond All In One Kerr™ (AIO), and Group 3 - Adper single bond 3M™ (ASB). The former two are self-etching adhesive bonding agents and the last one is a two-step adhesive. Group 4 corresponds to the control group without any applied adhesive but using a Bluephase N curing lamp (Ivoclar Vivadent). The application of adhesive agents on top of dentine discs was carried out according to the instructions supplied by the manufacturer, which are shown in Box 1.

Samples were measured by triplication and three tests were performed to evaluate cell viability: Raman Spectroscopy, confocal fluorescence microscopy, and MTT assay.

**Box 1:**
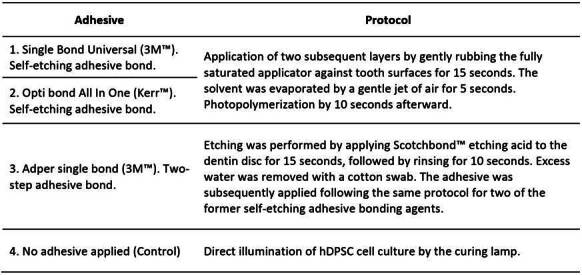
Protocol established by the manufacturers for the application of adhesives on dentine discs

### Procedures for Cell Viability Measurements

After seven days of cell culture and reaching a confluence higher than 80% (Figure 3), adhesives were applied to dentine discs and subsequently photopolymerized. Following 24 hours of exposure to the adhesive, dentine discs are dismounted and cell viability of hDPSC is evaluated through Raman Spectroscopy, confocal fluorescence microscopy, and MTT assay, as described:

- *Raman Spectroscopy.* hDPSC sewn on SERS substrates were examined using an Ocean Optics IDR-Micro 785 Raman spectrometer, which has a 40X optical microscope that allows focusing the 785 nm laser beam of 10 micrometers diameter on individual cells. Measurements were carried out with 2.1 mW optical power for 15 seconds and 10 scans, to prevent structural damage to the cells.
- *Confocal fluorescence microscopy*. Sample were prepared according to the instructions supplied with Invitrogen™ LIVE/DEAD™ Viability/Cytotoxicity Kit. Before its application, cells were rinsed with PBS to remove debris. For kit preparation 10 mL of PBS, 5 µL calcein, and 20 µL Ethidium homodimer were mixed in a Falcon tube. Calcein is absorbed by esterase, an active enzyme in alive cells with green photoluminescence. Ethidium homodimer is absorbed by nucleic acids on cells with plasmatic membranes broken and red fluorescence. 150 µL of the mix was added to the cell culture and kept for 30 minutes in darkness at room temperature for their observation under the confocal microscope. Quantification of live and dead cells was assisted by Image J software ^26^.
- *MTT assay.* A solution of 450 mL DMEM medium containing 0.2 mg/mL MTT salt (3-(4,5-Dimethylthiazol-2-yl)-2,5-Diphenyltetrazolium Bromide), 50 mL SFB, 5 mL Penicillin and 5 mL Glutamine was prepared. MTT salt is dissolved by shaking. Then, 100 µL of this solution is added to the cell culture after the medium has been aspirated. Incubation lasts 4 to 7 hours at 37ºC under an atmosphere of 5% CO_2_ and 95% humidity. Monitoring of the characteristic violet color of formazan crystals is performed. Afterwards, formazan is dissolved when removing the medium with MTT salt and adding 100 µL dimethyl sulfoxide. The microplate is then introduced into the absorbance reader at the chosen 570 nm wavelength. Based on cell concentration, cytotoxicity is then estimated according to the following criteria: Non-cytotoxic (cell viability higher than 75%), slightly toxic (50% to 74%), moderately (25% to 49%), and extremely toxic for cell viability lower than 24%.

## Results

### Penetration of adhesives into dentine microtubules

**Figure 5:**
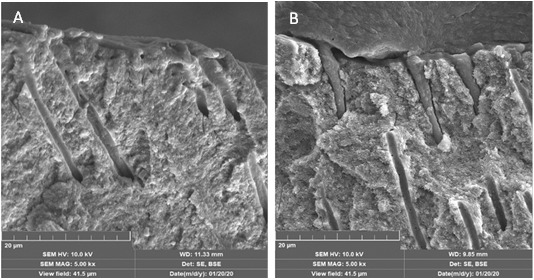
SEM images at the dentine/adhesive interface. a) Self-etching adhesive Single Bond Universal 3M™. b) Two-step adhesive Adper single bond 3M™.

SEM images of the interface between dentine discs and the polymerized adhesives were taken to explore the degree of penetration of adhesive into dentine microtubules. Figure 5a shows that self-etching adhesive Single Bond Universal 3M™ remains on the disc surface i.e., it has minimal penetration (minitags). This is not the case for Adper single bond 3M™ where tags of about 20 microns are observed (Figure 5b), suggesting an increased probability of reaching cell culture for this kind of two-step adhesive.

### Raman Spectroscopy Analysis

**Figure 6:**
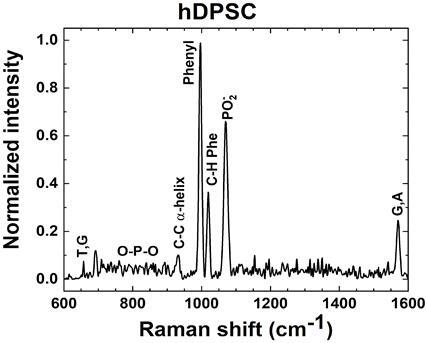
Representative normalized Raman Spectrum of hDPSC

Raman spectra of individual hDPSC grown on SERS substrates were taken as reference (control), and their maximum intensity peak was normalized to the unity (Figure 6).

The most intense band is assigned to the symmetric breathing mode of Phenyl rings. This indexation is supported for the identification of in-plane C-H modes in the Phenyl ring. There is another prominent band associated with the stretching mode of phosphodioxy group PO_2_^-^ on DNA. Nucleobases such as thymine, guanine, and adenine can be also identified, as well as the stretching mode of C-C in the α-helix. ^27^.

**Figure 7:**
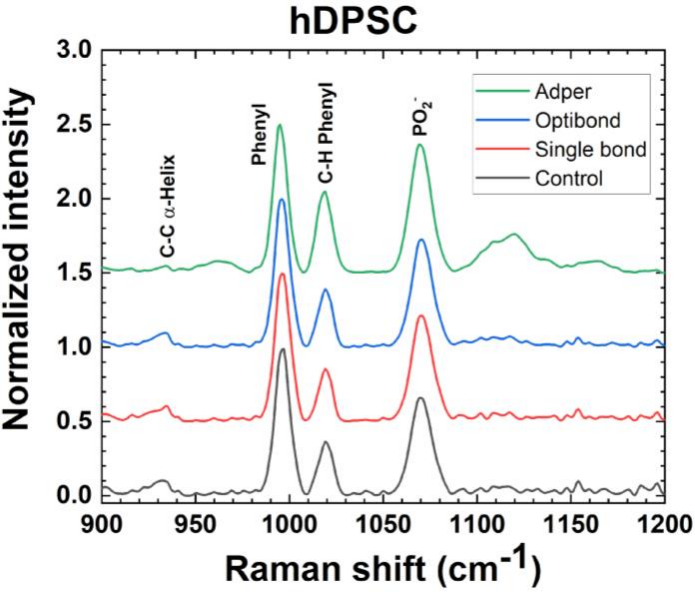
Raman spectra of hDPSC a) with no adhesive applied (control) and under exposure to the adhesives: b) Single bond universal, c) Optibond all in one, and d) Adper single bond.

Raman spectra of different hDPSC exposed during 24 hours to three different adhesives with a dentine barrier of 1.0 mm are compared to those without any exposure (control) in Figure 7.

The spectra shown here are the average of a complete set reported in the supplementary information (Figure S1). For the sake of clarity, only the more prominent Raman bands are shown. The relative intensity of the peaks looks quite similar for the two self-etching adhesive bonding agents (Single Bond Universal 3M™ and Opti bond All In One Kerr™). Instead, significant changes are observed for the two-step adhesive (Adper single bond 3M™).

**Figure.8. Renormalized:**
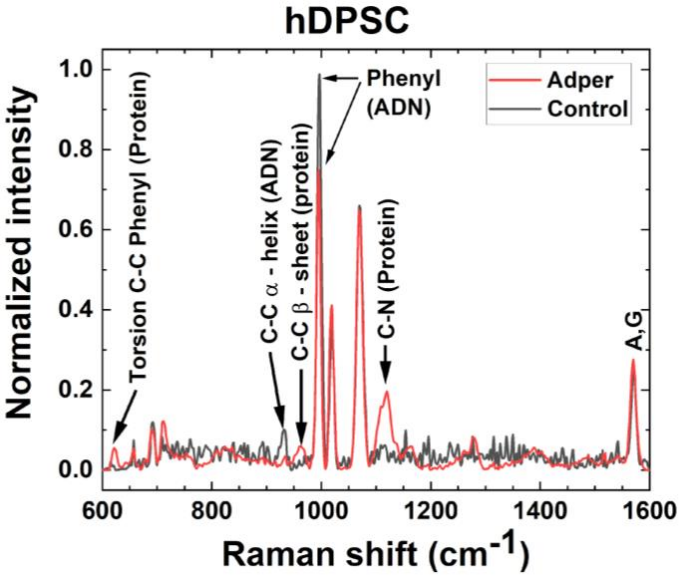
spectra of the two-step adhesive Adper single bond 3M™ and the control spectrum taking the Adenine, Guanine band as reference.

In Table 1, are estimated the relative intensities of the more intense Raman bands illustrated in Figure 8, taking into account that the maximum intensity associated with the breathing mode of the Phenyl ring has been normalized to one. Table 1 also includes the intensity of the Raman peak corresponding to Guanine and Adenine nucleobases located at 1580 cm^-1^.

**Table 1:** Relative intensities of most prominent Raman bands of hDPSC after being exposed to three different adhesives

Adhesive exposure	Relative intensity of Raman Bands		
	C-H Phenyl	PO_2_^-^	G, A
No adhesive (Control)	0.39 ± 0.08	0.68 ± 0.11	0.29 ± 0.06
Single Bond Universal 3M™	0.36 ± 0.02	0.72 ± 0.03	0.26 ± 0.05
Opti bond All In One Kerr™	0.39 ± 0.02	0.72 ± 0.04	0.28 ± 0.04
Adper single bond 3M™	0.55 ± 0.02	0.86 ± 0.07	0.42 ± 0.15

Quantification of relative intensities allows us to conclude that there are no significant differences between the Raman spectra of self-etching adhesive bonding agents and those without exposure to adhesives. Therefore, no cytotoxic effects originating from the application of this type of adhesive are expected.

On the other hand, the increase of the relative intensities for all the bands observed for the two-step adhesive Adper single bond 3M™ can be attributed to a reduction in the intensity of the band assigned to the breathing mode of the Phenyl ring. To confirm this observation, a renormalization of this spectrum is proposed in Figure 8, taking now the GA band as a reference for comparison concerning the control spectrum.

Besides the reduced intensity of the Phenyl ring peak, the band corresponding to C-C in the α-Helix vanishes. Conversely, there is an increase in the bands assigned to the stretching mode of C-C bonds in β-sheet, the stretching mode of C-N bonds, and the torsion mode of C-C bonds in Phenyl, respectively. Altogether, these findings suggest possible cytotoxic effects affecting the α-Helix DNA structure and leaving their β-sheet and nitrogenous bases more exposed, and even introducing more degrees of freedom for non-bonded phenyl rings.

### Cell viability by confocal fluorescence microscopy and MTT assay

**Figure 9:**
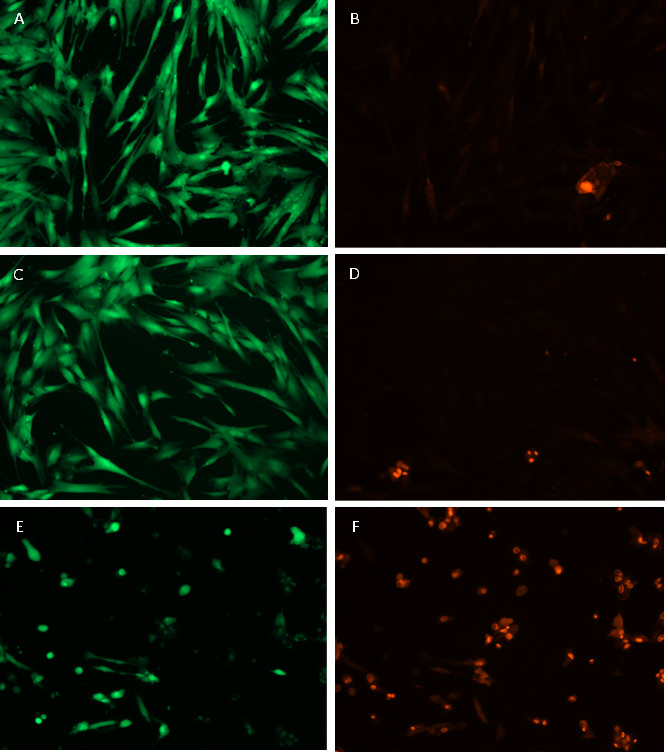
Images of Fluorescence emitted by hDPSC after exposure to adhesives: Single bond universal (a,b), Optibond all in one (c,d), and Adper single bond (e,f).

Fluorescence microscopy images were taken with an objective 10X/0.30 (U Plan FL. N) using the Live/Dead kit on hDPSC after exposure to the three adhesives (Figure 9). Green fluorescence emitted by Calcein is associated with alive cells and the red one, correlated to dead cells, stems from ethidium bromide. 

At a glance, cell viability is significantly higher for self-etching adhesive bonding agents than the two-step adhesive Adper single bond, in agreement with the Raman analysis previously done. Quantitative results are summarized in Table 2, taking 100% reference to a single fluorescence image of hDPSC without exposure to any adhesive. The significance of the data is demonstrated by the ANOVA test in the supplementary information.

**Table 2:** Summary of cell viability estimated by MTT test and confocal fluorescence microscopy.

Adhesive	Method		
	Fluorescence	MTT assay	Total
No adhesive (Control)	97.3 ± 2.8%	104.9 ± 5.3%	102.8 ± 5.8%
Single Bond Universal 3M™	98.9 ± 1.1%	102.0 ± 9.1%	100.1 ± 6.0%
Opti bond All In One Kerr™	93.6 ± 2.7%	100.5 ± 6.6%	97.7 ± 6.4%
Adper single bond 3M™	95.3 ± 3.3%	101.4 ± 6.4%	98.9 ± 6.2%

## Discussion

Restorative procedures performed in oral cavities can affect pulp tissue depending on cavity preparation and materials employed in the procedure. The most effective protection for the pulp is the dentine itself, which improves when increasing thickness. In the present study, 1 mm thick dentine discs were used to simulate deep cavities, where the use of self-etching adhesive agents is indicated. Although the dentine barrier simulates the clinical situation, there are still several factors influencing the diffusion of substances into dentine. Permeability varies among individuals due to structural differences and composition and can provide different results depending on their location ^28^. For the present work, all dentine discs were extracted from third molars with standard cuts at very a specific zone. Considering that teeth come from different patients, variations in their structure and composition are unavoidable. Despite this fact, the obtained results have low dispersion as can be seen in Table 1.

The interaction of self-etching adhesive with pulp tissue is a key issue for their success. Therefore, human dental stem pulp cells (hDSPC) were used to simulate this interaction in clinical practice when self-etching adhesives are applied in deep cavities. Three different adhesive agents were tested for toxicity of hDSPC, via cell viability. Two of them are self-etching adhesives, and the other one is a two-step adhesive agent.

Cell viability was assessed by Raman Spectroscopy and their results were contrasted with those provided by Confocal fluorescence microscopy and MTT assay. Table 2 showed cell viability higher than 97% when exposed to self-etching adhesives with a dentine barrier of at least 1 mm thickness. Raman spectroscopy results summarized in Figure 8 and Table 1, exhibit DNA, and RNA bands of hDSPC after 24 hours of exposure to self-etching adhesives with dentine barrier, having quite similar intensities of cells without any exposure to adhesives (Control). Instead, the Raman spectrum of the two-step adhesive had a significant reduction in Phenyl and α-helix bands associated with DNA and even the presence of new ones ascribed to β-sheet and new oscillation modes of Phenyl rings (Figure 9). These findings reveal structural damage to DNA α-helix in cells (Red color fluorescence in Figure 10), suggesting that this kind of adhesive could cause damage to pulp tissue when applied to deep cavities in clinical practice. This could be attributed to the removal of the smear layer by orthophosphoric acid or its penetration through dentine microtubules, among other factors. Keeping the smear layer according to the protocol of this study, as illustrated in Figure 2, supports their role in preventing the diffusion of residual monomers to pulp cells and promoting adhesion.

The two self-etching adhesives used in this study have shown non-significant cytotoxicity levels, suggesting that the current protocol indicated for adhesion deep dentine cavities is quite safe for clinical practice. This agrees with previous studies considering Single Bond Universal 3M™ as a moderate self-etching adhesive (pH 2.7), that causes partial demineralization of dentine leaving hydroxyapatite residuals attached to collagen, allowing chemical adhesion to dihydrogen methacryloxydocylphosphate (MDP) ^29^ ^,^ ^30^. Other studies pointed out to 30-50% reduction in cell viability after using self-etching adhesives in deep cavities. However, in that case, permeation of residual monomers to pulp cells was demonstrated, due to the use of thinner dentine barriers (0.4 mm) and remotion of the smear layer by EDTA ^28^ ^,^ ^31^.

Conversely, adhesion in two-step agents depends mainly on resin tags infiltrated within dentinal microtubules and inter-tubular dentine. Infiltration and therefore adhesion is more difficult in deep dentine because inter-tubular dentine decreases when approaching the pulp, and dentinal microtubules increase their size and diameter, being more permeable to dentinal liquid. In addition, phosphoric acid at 37% is strong enough to remove the smear layer, leaving microtubules with fluid more exposed, and hindering tag formation ^25^ ^,^ ^28^. In this study, however, tag formation in 1 mm thick dentine discs is observed in Figure 6. This is probably owing to the absence of intratubular liquid, despite dentine discs being damp after being immersed into the culture medium. Tag formation provides a barrier for the diffusion of residual monomers.

No dramatic reduction of cell viability was observed in this study when using 1 mm thick dentine barriers. Decreased cell viability was reported previously for the total-etch adhesive technique using thin dentine barriers of 0.4 mm, which seems to confirm the relevance of dentine thickness for preventing cytotoxic effects. Galler *et al.* demonstrated that 0.5 mm thick dentine barriers can shelter 75% of pulp tissue from toxic agents, rising to 90% for 1 mm thick barriers and causing just tiny damages for dentine barriers of 2 mm thickness ^28^ ^,^ ^31^ ^,^ ^32^. According to this, the results obtained in the present study suggest that the use of both types, self-etching and two-step adhesives, is safe when leaving dentine barriers of at least 1 mm thickness. Nevertheless, self-etching adhesives are recommended for better results, especially when dealing with deep cavities. 

Cell viability through fluorescence microscopy and MTT assay was evaluated in the frame of this work. These are standard and reliable tests that have shown similar results for the three adhesive agents. In addition, the potential of Raman spectroscopy as a novel technique for assessing cytotoxic effects due to the employment of dental materials was tested ^33^.

Although challenging, the elaboration of SERS substrates allowed us to investigate the influence of adhesives on individual hDPSC, providing a closer look at cytotoxic effects on the inner structure of cells beyond cell viability. That means Raman spectroscopy is probably not a valuable tool for estimating cell viability, but it is a very powerful technique able to identify structural damage of individual cells and therefore, has a huge potential to evaluate cell cytotoxicity in the full meaning of the word. The scientific challenge is to make this potential by unambiguously identifying the origin of measured Raman bands, which could enable their use as a standard technique for diagnosis in clinical practice.

## Conclusions

Raman spectroscopy has shown its capability to reveal structural DNA changes in the inner structure of some cells when a two-step adhesive is applied. Chemical components of self-etching adhesives do not have significant cytotoxic effects on human dental pulp stem cells on dentine barriers of at least 1 mm.
